# Development and validation of a predictive model of the impact of single nucleotide polymorphisms in the *ICAM-1* gene on the risk of ischemic cardiomyopathy

**DOI:** 10.3389/fcvm.2022.977340

**Published:** 2022-11-10

**Authors:** Tuersunjiang Naman, Refukaiti Abuduhalike, Mubalake Yakufu, Ayixigu Bawudun, Juan Sun, Ailiman Mahemuti

**Affiliations:** Department of Heart Failure, First Affiliated Hospital of Xinjiang Medical University, Urumqi, China

**Keywords:** gene polymorphism, variants of ICAM-1 gene, ischemic cardiomyopathy (ICM), predictive model, risk factors of ICM

## Abstract

**Objective:**

Previous research has linked single nucleotide polymorphisms (SNPs) in the *ICAM-1* gene to an increased risk of developing ischemic cardiomyopathy (ICM); however, a diagnostic model of ICM according to the *ICAM-1* variant has not yet been developed. Therefore, this study aimed to explore the correlation between SNPs in *ICAM-1* and the presence of ICM, along with developing a diagnostic model for ICM based on the variants of the *ICAM-1* gene.

**Method:**

This study recruited a total of 252 patients with ICM and 280 healthy controls. In addition, all the participants were genotyped for SNPs in the *ICAM-1* gene by polymerase chain reaction-restriction fragment length polymorphism (PCR-RFLP). Using the training dataset of 371 people, we constructed a nomogram model based on *ICAM-1* gene variants and clinical variables. To optimize the feature choice for the ICM risk model, a least absolute shrinkage and selection operator (LASSO) regression model was adopted. We also employed multivariable logistic regression analysis to build a prediction model by integrating the clinical characteristics chosen in the LASSO regression model. Following the receiver operating characteristic (ROC), a calibration plot and decision curve analysis (DCA) were used to evaluate the discrimination, calibration, and clinical usefulness of the predictive model.

**Result:**

The predictors involved in the prediction nomogram included age, smoking, diabetes, low-density lipoprotein-cholesterol, hemoglobin, N-terminal pro-B-type natriuretic peptide, ejection fraction, and the rs5491 SNP. The nomogram model exhibited good discrimination ability, with the AUC value of ROC of 0.978 (95%CI: 0.967–0.989, *P* < 0.001) in the training group and 0.983 (95% CI: 0.969–0.998, *P* < 0.001) in the validation group. The Hosmer–Lemeshow test demonstrated good model calibration with consistency (*P*_training group =_ 0.937; *P*_validation group =_ 0.910). The DCA showed that the ICM nomogram was clinically beneficial, with the threshold probabilities ranging from 0.0 to 1.0.

**Conclusion:**

The AT genotype in rs5491 of the *ICAM-1* gene was associated with having a higher frequency of ICM. Individuals carrying the mutant AT genotype showed a 5.816-fold higher frequency of ICM compared with those with the AA genotype. ICM patients with the AT genotype also had a higher rate of cardiogenic death. We, therefore, developed a nomogram model that could offer an individualized prediction of ICM risk factors.

## Introduction

Cardiovascular diseases (CVDs) are one of the common causes of cardiogenic death worldwide (1). In particular, ischemic heart disease (IHD) is the prominent cause of morbidity and mortality globally (2), which most often results from coronary artery atherosclerosis. In the first National Health and Nutrition Examination Survey (NHANESI), coronary artery disease (CAD) accounted for < 60% of the incident cases of heart failure (HF), with hypertension and diabetes mellitus contributing 10 and 3%, respectively (3). Other vascular diseases may also impede blood flow to the cardiac tissue (4). The main cause of CVDs is reduced blood flow to the myocardium, resulting in heart muscle injury (5). In the United States, ischemic cardiomyopathy (ICM) is the major etiology of CVDs and the greatest risk factor for developing HF (6). According to the global pandemic estimate, approximately 26 million patients were affected by cardiac insufficiency, costing global health systems over $30 billion (7, 8). Moreover, the mortality rate of patients with cardiac disorders has also been higher than 50% in the last 5 years (9, 10). Therefore, a precise diagnostic method and therapeutic regimen must be developed to enhance the diagnosis and treatment of CVDs (11).

Atherosclerotic lesions in multi-coronary arteries, especially diffusive lesions, which can induce severe myocardial dysfunction, are the root cause of ICM (12). As shown in previous studies, the blood levels of intercellular adhesion molecule-1 (ICAM-1) are recognized to be a marker of coronary artery atherosclerosis and the progression of coronary heart disease (CHD) (1, 2, 13). As a member of the immunoglobulin superfamily, *ICAM-1* is highly expressed in endothelial cells and leukocytes. It plays the role of a receptor for the leukocyte integrin lymphocyte function-related antigen-1 and Mac-1 (3–5). *ICAM-1* is a vital factor in the pathogenic mechanism of atherosclerosis and has a significant function in recruiting mononuclear cells into the basement membrane of the vasculature (6, 7). Thus, *ICAM-1* can play a major role in the development of atherosclerosis and ICM.

Although researchers found a correlation between the ICAM-1 gene and atherosclerosis or CVDs, we don't have sufficient evidence to prove that the polymorphism of the ICAM-1 gene variants correlates with ICM. Furthermore, a diagnostic model for ICM according to *ICAM-1* polymorphisms has not yet been developed. Therefore, the present study attempted to identify the connection between polymorphisms in the *ICAM-1* gene (rs112872667, rs12462944, rs2358581, rs281430, rs281434, rs3093030, rs3093032, rs5030348, rs5030377, rs5491, rs62130660, and rs923366) and ICM in patients with ICM in comparison with control subjects. We also aimed to establish a novel nomogram model to diagnose ICM more accurately based on polymorphisms of the *ICAM-1* gene.

## Materials and methods

### Ethical approval of the study protocol

Before starting this study, all participants signed the informed consent form. The standards of the Declaration of Helsinki were applied to design the study. This study was approved by the Ethics Committee of the First Affiliated Hospital of Xinjiang Medical University.

### Study design and populations

**Figure 1:**
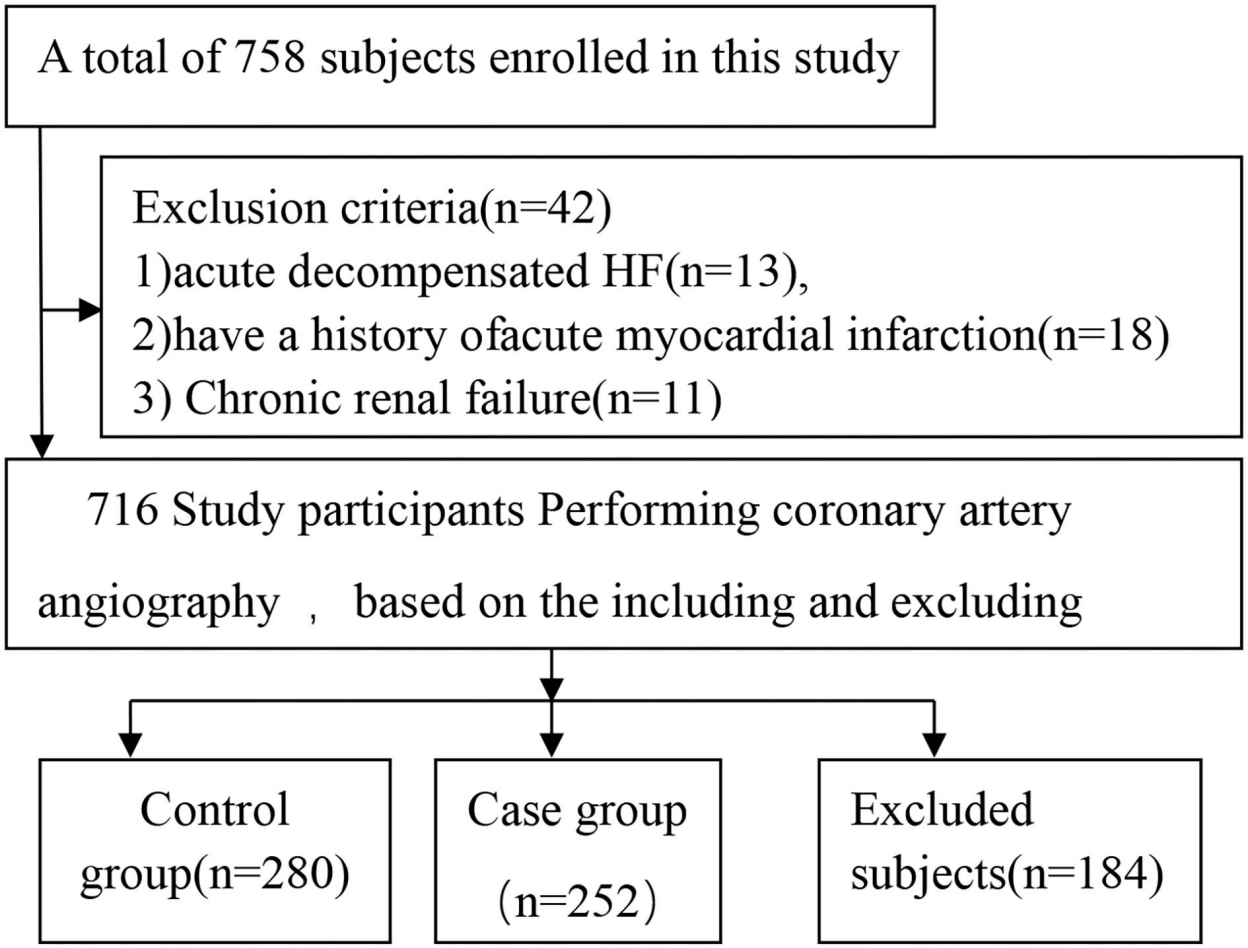
Roadmap of screening and grouping the study cohorts.

The participants were enrolled at the First Affiliated Hospital of Xinjiang Medical University between January 2013 and December 2015. A total of 758 participants were enrolled in this study. After applying the inclusion and exclusion criteria, we finalized 532 participants; of those, 252 were ICM patients, and 280 were control subjects (Figure 1). All study subjects had previously undergone coronary angiography in the hospital or during their last hospitalization.

ICM patients were diagnosed according to the following criteria: (1) coronary angiography (confirmed by at least two experienced cardiologists) revealed luminal stenosis larger than 50% in one or more of the coronary arteries of the leading branch or a history of performed percutaneous coronary intervention or coronary artery bypass grafting (2) impaired myocardial function as characterized by N-terminal pro-B-type natriuretic peptide (NT-proBNP)>125 ng/mL; (3) predictable angina that could be relieved by rest or nitroglycerin; and (4) symptoms of chest tightness, shortness of breath, or dyspnea that were usually promptly relieved by rest.

Exclusion criteria included the following: acute decompensated HF; a history of acute myocardial infarction; unstable hemodynamics; hepatic, nephritic, hematologic, or autoimmune disorders; non-cardiac disease with an expected survival of < 1 year; cachexia; and unwillingness to participate. The control group included patients with (1) < 50% luminal stenosis in the coronary artery, as confirmed by coronary angiography (diagnosed by at least two experienced cardiologists) and (2) absence of angina on exertion.

### Blood collection and laboratory tests

We obtained blood samples from all ICM patients and control subjects on the first day of hospitalization. The blood examination was performed in the laboratory of the First Affiliated Hospital of Xinjiang Medical University. In addition, the following parameters were tested: hemoglobin, white blood cell (WBC), platelet (PLT), creatinine (CR), blood urea nitrogen (BUN), high-and low-density lipoprotein-cholesterol (HDL-C and LDL-C, separately), triglyceride (TG), and total cholesterol (TC).

### DNA extraction

After laboratory testing, DNA was extracted from the venous blood samples. The blood samples were centrifuged with the anticoagulant ethylene diamine tetra acetic acid (EDTA) at 1500 rpm for 10 min to separate plasma and blood cells using an Eppendorf high-speed centrifuge. The whole-blood genome extraction kit (Xiamen Kaishuo Biotechnology Corporation, China) was used to extract DNA from peripheral leukocytes in accordance with the specific instructions of the manufacturer. In addition, the extracted DNA was maintained at −80 °C until genotyping analysis.

### Genotyping of the ICAM-1 gene

PCR was performed by DNA amplification (1 μL) following the manufacturer's instructions. SNP genotyping was carried out on the amplified samples using a SNaPshot multiplex SNP genotyping kit (Application Binary Interface Company, USA) in accordance with the manufacturer's instructions.

### Definition of cardiovascular risk factors

By dividing body weight (kg) by the square of body height (m), body mass index (BMI) was computed. Participants who smoked for the last 6 months or more than 6 months were considered smokers. Participants who had consumed at least 100 g of alcoholic beverage every week within the past month were considered drinkers. Based on the 2018 European Society of Cardiology (ESC)/European Society of Hypertension (EHS) Guidelines (14), hypertension was determined as follows: systolic blood pressure (SBP) ≥140 mmHg or diastolic blood pressure (DBP) ≥90 mmHg or application of antihypertensive medications within the last 2 weeks. Diabetes mellitus (DM) was diagnosed according to fasting plasma glucose levels of ≥7.0 mmol/L (126 mg/dL) or glucose levels of ≥11.1 mmol/L (200 mg/dL) 2 h after administering a 75 g oral glucose load, history of diabetes, or history of anti-diabetic medication usage.

### Study endpoints during the follow-up period

During hospitalization and after discharge, cardiogenic death was considered the endpoint of this study. Follow-up was performed by a telephonic interview with patients or their relatives. Information regarding patients who had died was acquired from hospital records or through telephonic discussions with the family members of the deceased. A telephonic interview was performed 3, 6, 12, 24, and 60 months after ICM diagnosis. Trained investigators performed the follow-up work, and three experienced researchers conducted data entry to ensure the quality of the data. For the follow-up work, a group of clinical physicians was trained to systematically obtain appropriate data and confirm all events.

### Statistical analyses

Data analyses were performed using SPSS 25.0, R 4.0.0, and Stata 15.0 software. Continuous data were denoted as mean with SD when normally distributed or medians with inter-quartile range when non-normally distributed and contrasted with Student's unpaired *t*-test or with the Mann–Whitney U-test. Categorial variables were denoted as numbers with proportions and were compared with the chi-square test.

**Figure 2:**
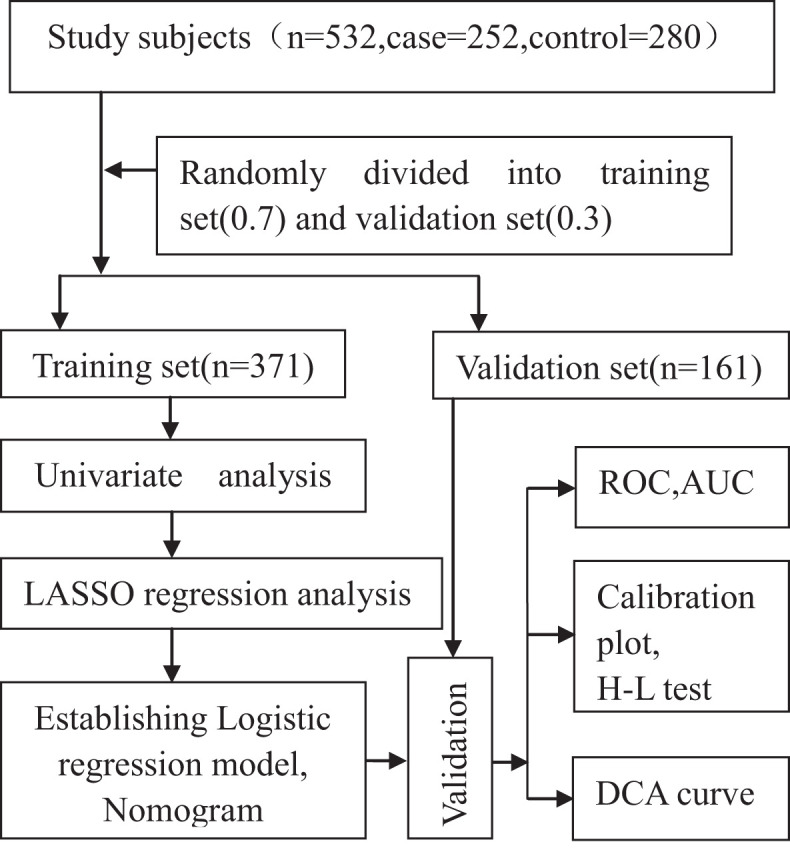
Roadmap of establishing and validating the model.

We compared the frequencies of genotypes in the control group with values expected by the Hardy–Weinberg equilibrium by adopting the chi-square test. All the study participants were randomly categorized into the following two groups, with 371 (70%) in the training group of the model and 161 (30%) in the validation group (Figure 2). The training group was divided into a case group (*n* = 181) and a control group (*n* = 190). We performed a univariate analysis in the training group. The optimal predictive features were selected using the least absolute shrinkage and selection operator (LASSO) method, which can adjust for the decrease in high-dimensional data (15, 16) by incorporating significant variables (*P* < 0.1) in a univariate analysis. This study chose features with non-zero coefficients in the LASSO regression model (17).

Subsequently, multivariable logistic regression analysis was adopted for developing a predictive model by incorporating variables chosen by the LASSO regression model. Moreover, the characteristics included β, SE, and odds ratio (OR) with a 95% confidence interval (CI) and a significant *P*-value. Two-sided statistical significance levels were determined, and the model was further optimized using the Akaike Information Criterion (AIC) value of the model. This study constructed an ICM nomogram using multivariable logistic regression analysis to offer the clinician a quantitative tool for predicting the individual probability of ICM.

Using the Kaplan-Meier method, we plotted the survival curve and calculated the *P*-value. The hazard ratio was calculated by Cox regression analysis.

### Validation of the model

With the use of the validation data set of 161 subjects, internal validation was conducted. The nomogram model was validated by discrimination ability, calibration ability, and clinical effectiveness. Initially, the receiver operating characteristic (ROC) curve was mapped to determine the discrimination ability. An area under the curve (AUC) value of 0.5 to 1.0 was considered, with a value closer to one suggesting the good performance of the predictive model (18). Second, to assess the accuracy of predictive ability and to determine the agreement between the predicted and observed severity, calibration plots were developed. In addition, the 45° diagonal line in the curve indicated good calibration with the perfect predictive ability for the true risk of the disease. The calibration plot and the Hosmer–Lemeshow test were adopted to assess the calibration ability (19). A decrease in the **χ^2^** value indicates an increase in the relevant *p*-value, which shows that the model will have better calibration. If there is a statistically significant difference (*P* < 0.05) between the predicted value of the model and the actual observed value, the model has poor calibration (19). Third, in order to determine the clinical usefulness of the ICM nomogram based on its net benefits at varying threshold probabilities, a decision curve analysis (DCA) was performed (20). In addition, the net benefit was also measured by subtracting the percentage of false-positive patients from the percentage of true-positive patients and then weighing the relative risk of giving up interventions compared to the negative outcome of unnecessary interventions.

## Results

A total of 758 participants were enrolled in this study. After applying the inclusion and exclusion criteria, we decided to proceed with 532 participants in the current study. Of these, 252 were ICM patients, and 280 were control subjects (Figure 1). The distribution frequency of every genotype of all SNPs of the *ICAM-1* gene in the control group was consistent with the Hardy–Weinberg equilibrium test. This indicated that the control subjects were representative of the study population and could be included in the present work.

All the study participants were categorized randomly into the following two groups, with 371 (70%) in the training group of the model and 161 (30%) in the validation group (Figure 2). By performing the Kolmogorov–Smirnov test in the training group, both the case group and control group, we found that all of the continuous variables were non-normal. Thus, we presented them as medians with an inter-quartile range (Table 1). in the training group, a univariate analysis of the basic clinical parameters between the case and control groups showed a significant age difference (*P* < 0.001), gender (*P* < 0.001), smoking habits (*P* < 0.001), hypertension (*P* < 0.05), diabetes (*P* < 0.001), PLT (*P* < 0.001), hemoglobin (*P* < 0.05), alanine aminotransferase (ALT) (*P* < 0.05), HDL-C (*P* < 0.001), LDL-C (*P* < 0.001), NT-proBNP (*P* < 0.001), ejection fraction (*P* < 0.001), and left ventricular end-diastolic volume (LEVD) (*P* < 0.001). No significant differences could be found in BMI, drinking habits, WBC, aspartate aminotransferase (AST), creatinine (CR), BUN, TC, and TG (*P* > 0.05) (Table 1).

**Table 1:** Univariate analysis of the clinical data.

**Variable**	**Control (*n* = 190)**	**Case (*n* = 181)**	**Z/χ^2^**	***P*** **-value**
Age, years	49 (40–58)	63 (53.5–71)	−8.445	<0.001
Gender Male, *n (%)*	97 (51.1)	125 (69.1)	12.508	<0.001
Female, *n (%)*	93 (48.9)	56(30.9)		
BMI,kg/m^2^	25.47 (23.19–28.56)	26.08 (23.44–28.53)	−0.721	0.471
Smoking, *n (%)*	40 (21.1)	78 (43.1)	20.762	<0.001
Drinking, *n (%)*	22 (11.6)	25 (13.8)	0.418	0.518
Hypertension, *n (%)*	72 (37.9)	101 (55.8)	11.943	0.001
Diabetes, *n (%)*	9 (4.7)	61 (33.7)	50.802	< 0.001
WBC, 10^9^/L	6.31 (5.4–7.45)	6.78 (5.31–8)	−1.394	0.163
PLT, 10^9^/L	231 (196–269)	204 (173–257)	−3.731	<0.001
Hemoglobin, g/L	138 (128.75–150.25)	137 (126–147)	−2.527	0.012
AST, μg/L	20.5 (17.08–28)	20.4 (16–31)	−0.079	0.937
ALT, μg/L	20.3 (15.74–30.11)	19.2 (12.1–30)	−1.965	0.049
CR, μmol/L	73 (63–83)	74 (61–91)	−0.880	0.379
BUN, mmol/L	5.6 (4.9–6.8)	5.8 (4.8–7.4)	−0.310	0.756
TC, mmol/L	4.05 (3.67–4.51)	4.02 (2.46–5.83)	−0.932	0.351
TG, mmol/L	1.26 (0.84–2.12)	1.29 (0.9–2.02)	−0.726	0.468
HDL-C, mmol/L	1.33 (1.14–1.59)	1 (0.84–1.25)	−5.854	<0.001
LDL-C, mmol/L	1.86 (1.68–1.97)	2.34 (1.6–2.91)	−3.810	<0.001
NT-proBNP, ng/L	43 (18.8–390.25)	1306 (277–3364)	−11.334	<0.001
Ejection fraction, %	60 (44–65)	39 (35–44)	−12.495	<0.001
LVED, mm	48 (42–50)	54 (39.5–62.5)	−4.800	<0.001

The univariate analysis of SNPs between the case and control groups showed a significant difference in rs12462944 (*P* < 0.05), rs2358581 (*P* < 0.05), rs281430 (*P* < 0.05), rs281434 (*P* < 0.05), rs5030348 (*P* < 0.05), and rs5491 (*P* < 0.001) (Table 2). The AT genotype in rs5491 was also a risk factor for ICM (*P* < 0.05), but no significant difference was noted in rs112872667, rs281437, rs3093030, rs3093032, rs5030377, rs62130660, and rs923366 (*P* > 0.05).

**Table 2:** Univariate analysis of SNPs in the *ICAM-1* gene.

**SNP**		**Control (*n* = 190)**	**Case (*n* = 181)**	**χ^2^**	***P*** **-value**
rs112872667					
Genotype	CC	133 (70)	113 (62.4)	3.852	0.146
	CT	49 (25.8)	63 (34.8)		
	TT	8 (4.2)	5 (2.8)		
Dominant model	CC	73 (38.4)	53 (29.3)	3.452	0.063
	CT+TT	117 (61.6)	128 (70.7)		
rs12462944					
Genotype	GG	73 (38.4)	53 (29.3)	9.748	0.008
	GC	92 (48.4)	82 (45.3)		
	CC	25 (13.2)	46 (25.4)		
Dominant model	GG	73 (38.4)	53 (29.3)	3.452	0.063
	GC+CC	117 (61.6)	128 (70.7)		
rs2358581					
Genotype	TT	39 (20.5)	19 (10.5)	9.096	0.011
	TG	83 (43.7)	76 (42)		
	GG	68 (35.8)	86 (47.5)		
Dominant model	TT	39 (20.5)	19 (105)	7.069	0.008
	TG+GG	151 (79.5)	162 (89.5)		
rs281430					
Genotype	AA	100 (52.6)	70 (38.7)	8.708	0.013
	AG	77 (40.5)	101 (55.8)		
	GG	13 (6.8)	10 (5.5)		
Dominant model	AA	100 (52.6)	70 (38.7)	7.274	0.007
	AG+GG	90(47.4)	111 (61.3)		
rs281434					
Genotype	AA	39 (20.5)	17 (9.4)	8.988	0.011
	AG	77 (40.5)	85 (47)		
	GG	74 (38.9)	79 (43.6)		
Dominant model	AA	39 (20.5)	17 (9.4)	8.966	0.003
	AG+GG	151 (79.5)	164 (90.6)		
rs281437					
Genotype	CC	142 (74.7)	128 (70.7)	0.897	0.638
	CT	42 (22.1)	45 (24.9)		
	TT	6 (3.2)	8 (4.4)		
Dominant model	CC	142 (74.7)	128 (70.7)	0.756	0.385
	CT+TT	48 (25.3)	53 (29.3)		
rs3093030					
Genotype	CC	75 (39.5)	90 (49.7)	4.840	0.089
	CT	90 (47.4)	66 (36.5)		
	TT	25 (13.2)	25 (13.8)		
Dominant model	CC	75 (39.5)	90 (49.7)	3.944	0.047
	CT+TT	115 (60.5)	91 (50.3)		
rs3093032					
Genotype	CC	167 (87.9)	149 (82.3)	2.397	0.302
	CT	21 (11.1)	30 (16.6)		
	TT	2 (1.1)	2 (1.1)		
Dominant model	CC	167 (87.9)	149 (82.3)	2.281	0.131
	CT+TT	23 (12.1)	32 (17.7)		
rs5030348					
Genotype	AA	54 (28.4)	32 (17.7)	7.313	0.026
	AG	98 (51.6)	98 (54.1)		
	GG	38 (20)	51 (28.2)		
Dominant model	AA	54 (28.4)	32 (17.7)	6.006	0.014
	AG+GG	136 (71.6)	149 (82.3)		
rs5030377					
Genotype	AA	86 (45.3)	82 (45.3)	1.050	0.591
	AG	83 (43.7)	73 (40.3)		
	GG	21 (11.1)	26 (14.4)		
Dominantmodel	AA	86 (45.3)	82 (45.3)	0.000	0.994
	AG+GG	104 (54.7)	99 (54.7)		
rs5491					
Genotype	AA	169 (88.9)	132 (72.9)	15.539	< 0.001
	AT	21 (11.1)	49 (27.1)		
rs62130660					
Genotype	TT	123 (64.7)	120 (66.3)	0.429	0.807
	TG	57 (30)	54 (29.8)		
	GG	10 (5.3)	7 (3.9)		
Dominant model	TT	123 (64.7)	120 (66.3)	0.100	0.752
	TG+GG	67 (35.3)	61 (33.7)		
rs923366					
Genotype	CC	55 (28.9)	41 (22.7)	2.008	0.366
	CT	92 (48.4)	93 (51.4)		
	TT	43 (22.6)	47 (26)		
Dominant model	CC	55 (28.9)	41 (22.7)	1.915	0.166
	CT+TT	135 (71.1)	140 (77.3)		

### Clinical characteristics

**Figure 3:**
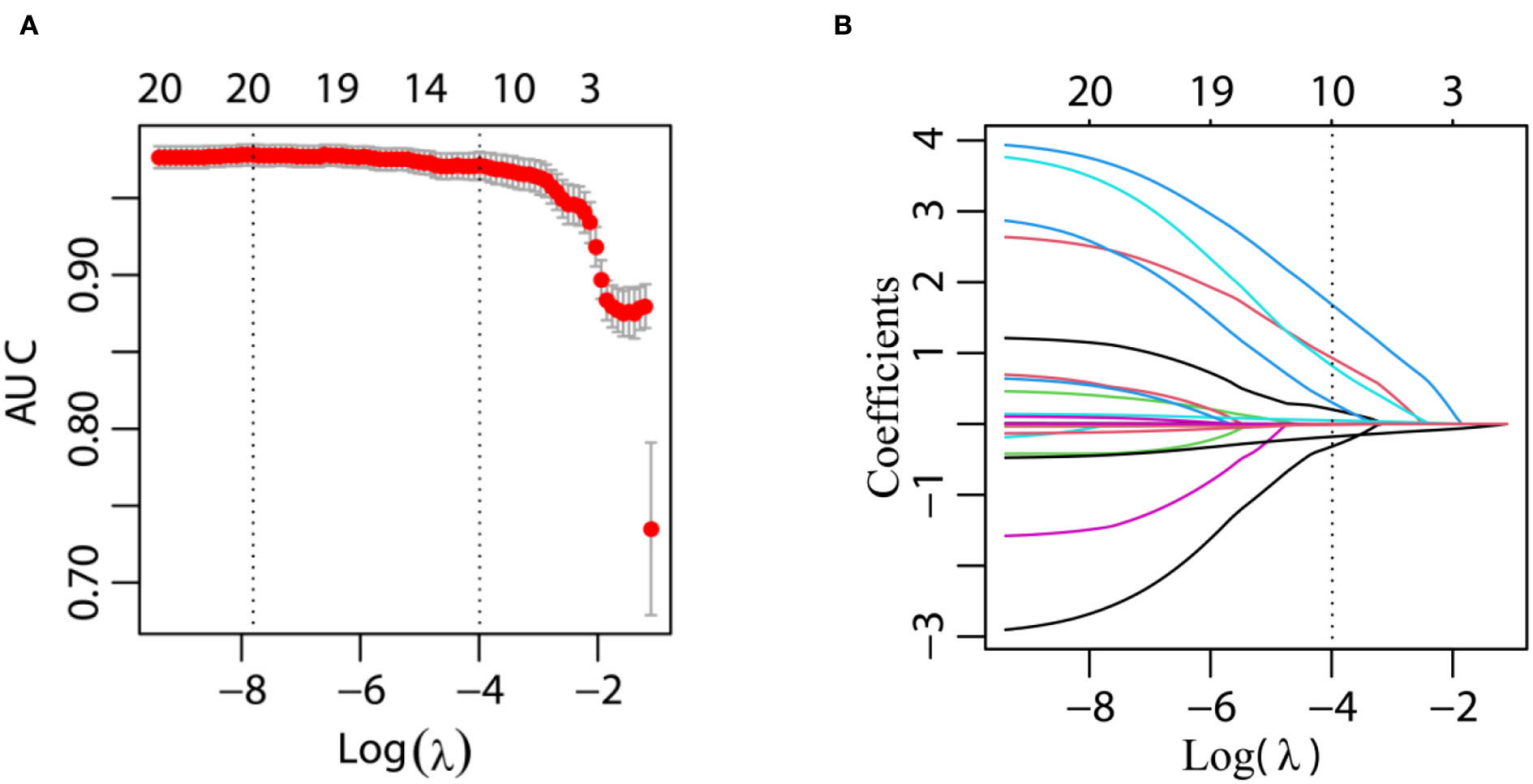
Variables selected by performing the LASSO binary logistic regression model. **(A)** Tuning parameter (λ) selection in the LASSO model employed 10-fold cross-validation through minimum criteria. Moreover, the AUC curve was mapped vs. log(λ). With the minimum criteria and the 1-standard error of the minimum criteria (the 1-SE criteria), the dotted vertical lines were plotted. A λ value of 0.018 was selected (1-SE criteria) in accordance with 10-fold cross-validation. **(B)** LASSO coefficient profiles of the 20 features. Obviously, a coefficient profile plot was constructed against the log(λ) sequence. We also drew a vertical line at the value chosen based on 10-fold cross-validation, in which the optimal λ value led to 10 non-zero coefficients.

From the univariate analysis of the clinical data and gene polymorphism characteristics, we incorporated 20 variables with *P* < 0.1 into the LASSO regression model, with the incorporation of the SNP variables based on the *P*-value of the dominant model. By examining the training group, which consisted of 371 participants, we were able to minimize the number of variables from 20 to 10 (Figures 3A,B). In addition, in the LASSO regression model, their coefficients were non-zero.

### Development of an individualized prediction model

We initially established a predictive model (Table 3, Model 1). The AIC value of this model was 154.087, with an AUC value of 0.979(95% CI: 0.967–0.989; *P* < 0.001). We then further established a simpler model (Table 3, Model 2) by optimizing Model 1 based on AIC value. The AIC value of Model 2 was 154.444, and the AUC value was 0.978 (95% CI: 0.967–0.989; *P* < 0.001). No significant difference was observed in either AIC or AUC value between Model 1 and Model 2 (*P* > 0.05); therefore, Model 2 was considered the best optimal model in the current work. The logistic regression analysis of the prediction model (Table 3, Model 2) showed that age, smoking, diabetes, LDL-C, hemoglobin, NT-proBNP, ejection fraction, and rs541 were independent risk factors for ICM and that those with a mutant AT genotype in rs5491 showed a higher frequency of ICM relative to those with the AA genotype (OR: 5.816, 95%CI: 1.661–20.362, *P* = 0.006).

**Table 3:** Parameters of the predictive model.

**Variable**	**Model1**					**Model2**			
	**β**	**SE**	**OR(95%CI)**	***P*** **-value**		**β**	**SE**	**OR(95%CI)**	***P*** **-value**
Age, years	0.100	0.022	1.106 (1.058–1.155)	<0.001		0.106	0.022	1.112 (1.066–1.159)	<0.001
Gender Male			1.000						
Female	−1.193	0.622	0.303 (0.09–1.026)	0.055					
Smoking	2.140	0.660	8.500 (2.330–31.008)	0.001		2.706	0.577	14.968 (4.831–46.381)	<0.001
Diabetes	3.238	0.695	25.485 (6.528–99.494)	<0.001		3.159	0.681	23.552 (6.205–9.393)	<0.001
LDL-C,mmol/L	2.829	0.624	16.925 (4.981–57.514)	<0.001		2.776	0.583	16.06 (5.122–50.355)	<0.001
Hemoglobin, g/L	−0.039	0.014	0.961 (0.935–0.989)	0.006		−0.029	0.013	0.972 (0.948–0.997)	0.027
NT-proBNP, ng/L	0.001	0.001	1.001 (1.000–1.002)	0.007		0.001	0.001	1.001 (1.000–1.002)	0.031
Ejection fraction,%	−0.368	0.087	0.692 (0.583–0.821)	< 0.001		−0.407	0.097	0.666 (0.551–0.805)	<0.001
rs3093030 CC			1.000						
CT+TT	−0.361	0.527	0.697 (0.248–1.960)	0.494					
rs5491 AA			1.000						
AT	1.843	0.667	6.313 (1.708–23.333)	0.006		1.761	0.639	5.816 (1.661–20.362)	0.006
Constant	8.616	4.026		0.032		6.401	4.268		0.134
AIC			154.087					154.444	
AUC(95% CI)			0.979 (0.969–0.990)					0.978 (0.967–0.989)	

**Figure 4:**
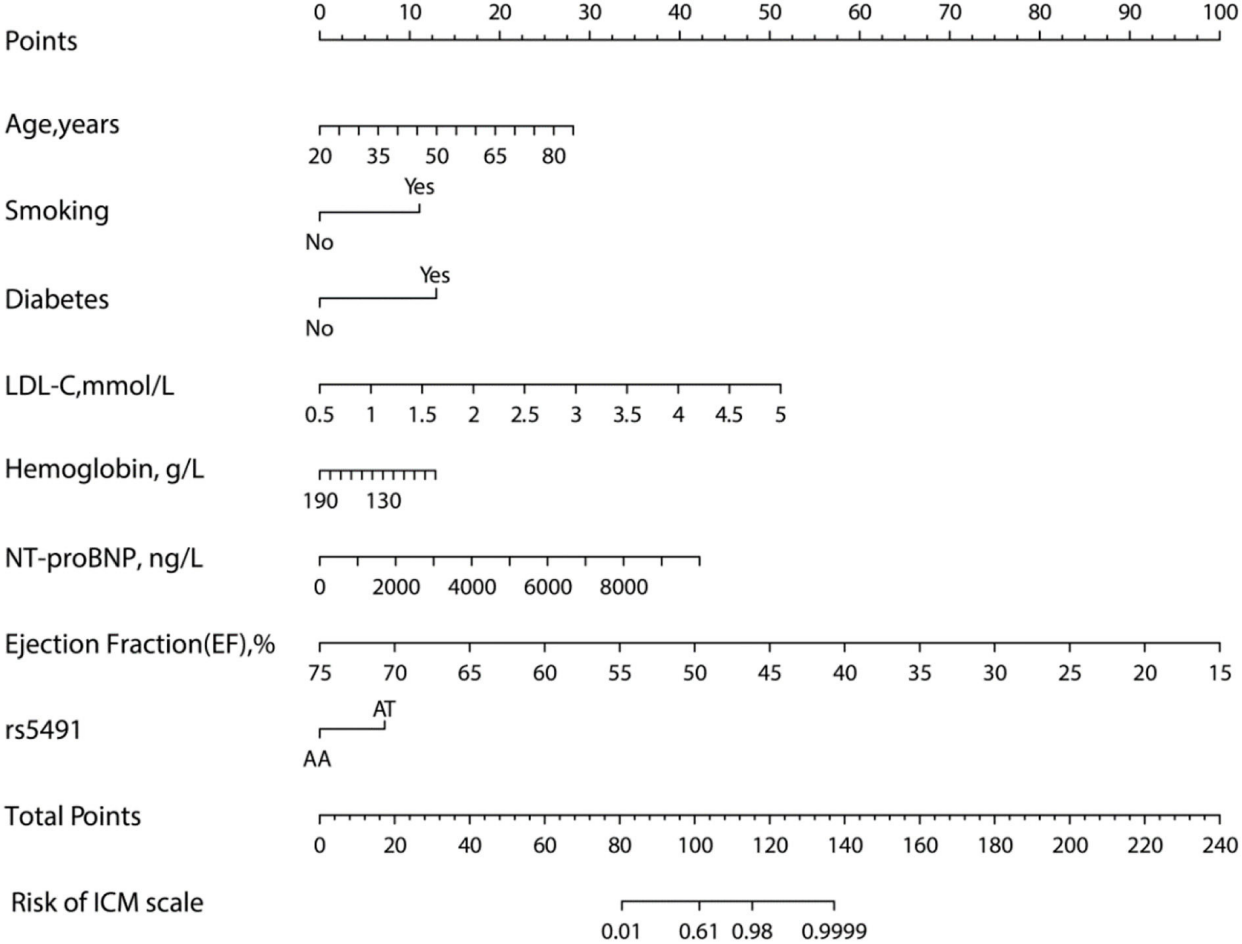
Nomogram for evaluating the risk of ICM. The nomogram was developed using several clinical and genomic variables, including age, smoking, diabetes, LDL-C, hemoglobin, NT-proBNP, ejection fraction, and rs5491 (figure generated by R software, https://www.r-project.org).

The model (Model 2) that included the above variables was built and constructed as a nomogram (Figure 4).

### Validation of the nomogram

**Figure 5:**
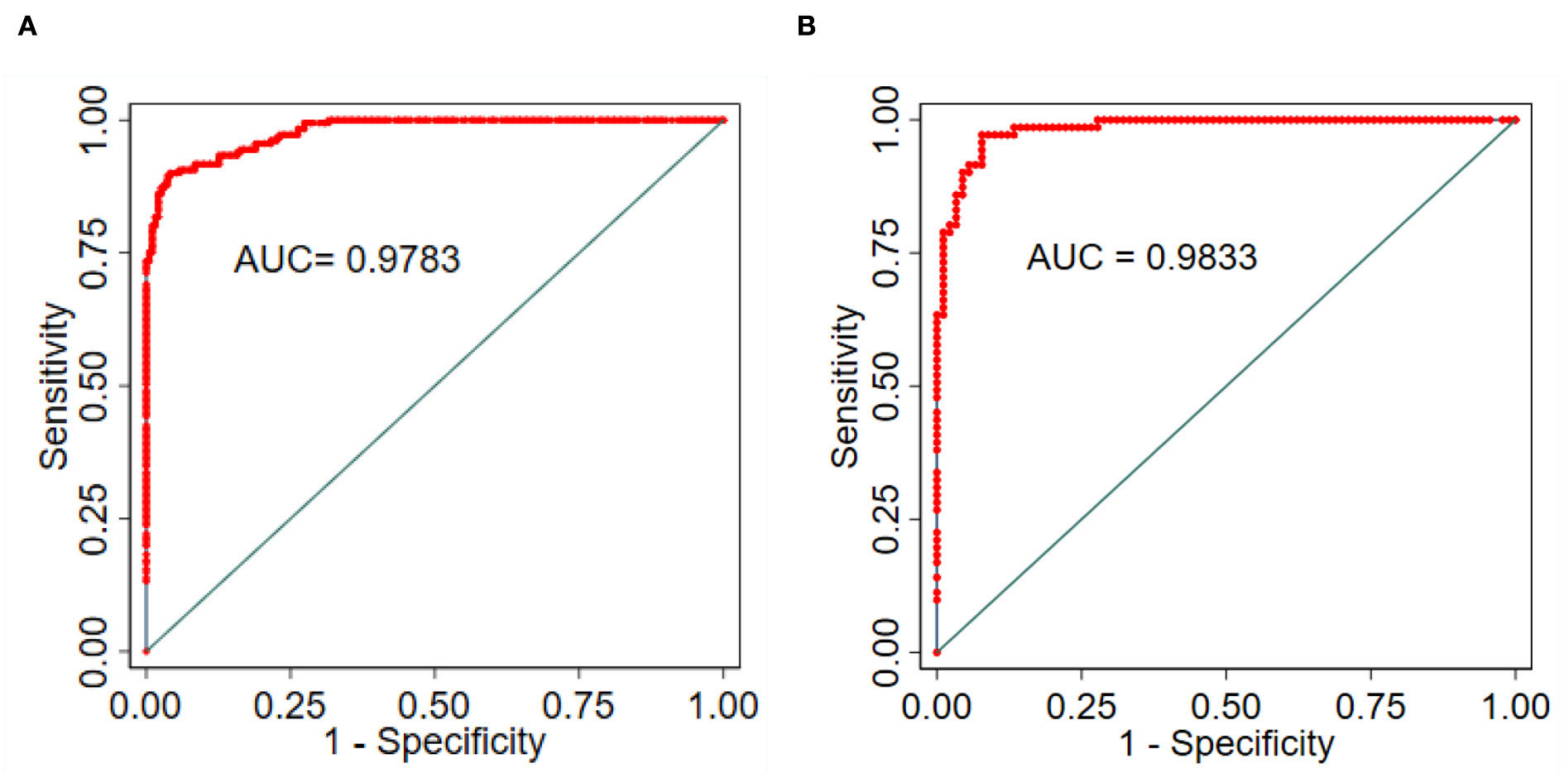
**(A)** Training group. **(B)** Validation group, and ROC to confirm the discrimination power of the nomogram.

The nomogram was validated based on its discrimination ability, calibration ability, and DCA in the training and validation groups. In addition, the predictive nomogram exhibited good discriminative ability, which can be seen in (Figures 5A,B). The AUC value of ROC was 0.978 (95% CI: 0.967–0.989; *P* < 0.001) in the training group and 0.983 (95% CI: 0.969–0.998; *P* < 0.001) in the validation group, which indicated that the model has good predictive power.

**Figure 6:**
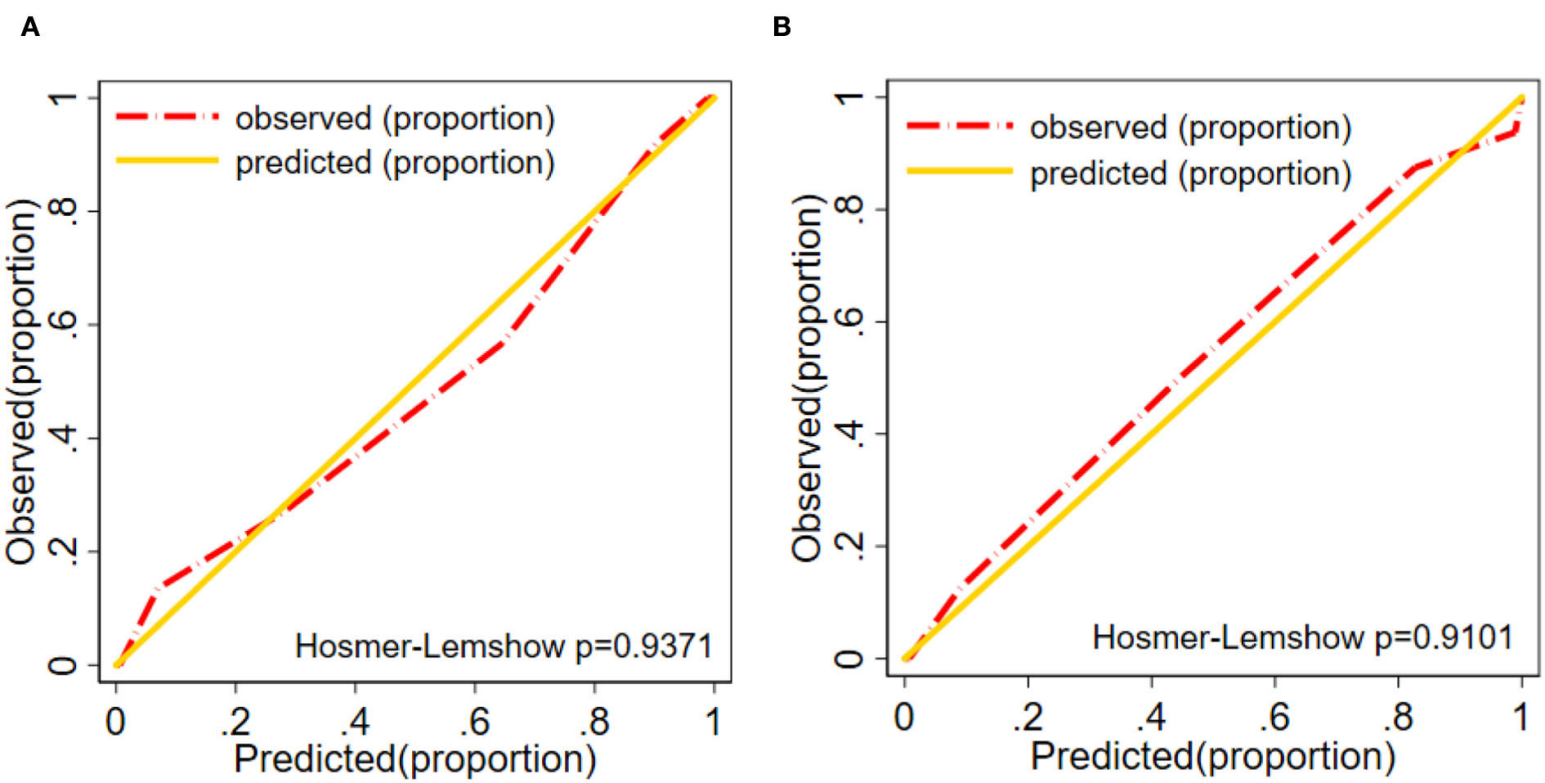
Calibration plot **(A)** Training group. **(B)** Validation group of the nomogram (*P*_training group =_ 0.937, *P*_validation group =_ 0.910). The diagonal gold line indicates a good prediction by the ideal model. In addition, the diagonal 45° gold line represents a good calibration where the model's predictive ability has high accuracy in indicating the actual risk of ICM. The red line shows the performance of the nomogram, and a closer fit to the diagonal red line indicates a good predictive value.

The ICM risk nomogram calibration plot revealed good agreement between the predicted value and the observed value in this cohort (Figures 6A,B). According to the Hosmer–Lemeshow test, there was a non-significant difference in the training group (*P* = 0.9371) and the validation group (*P* = 0.9101), thus indicating no departure from the perfect fit, which confirmed a high consistency between the predicted probability and the actual probability.

**Figure 7:**
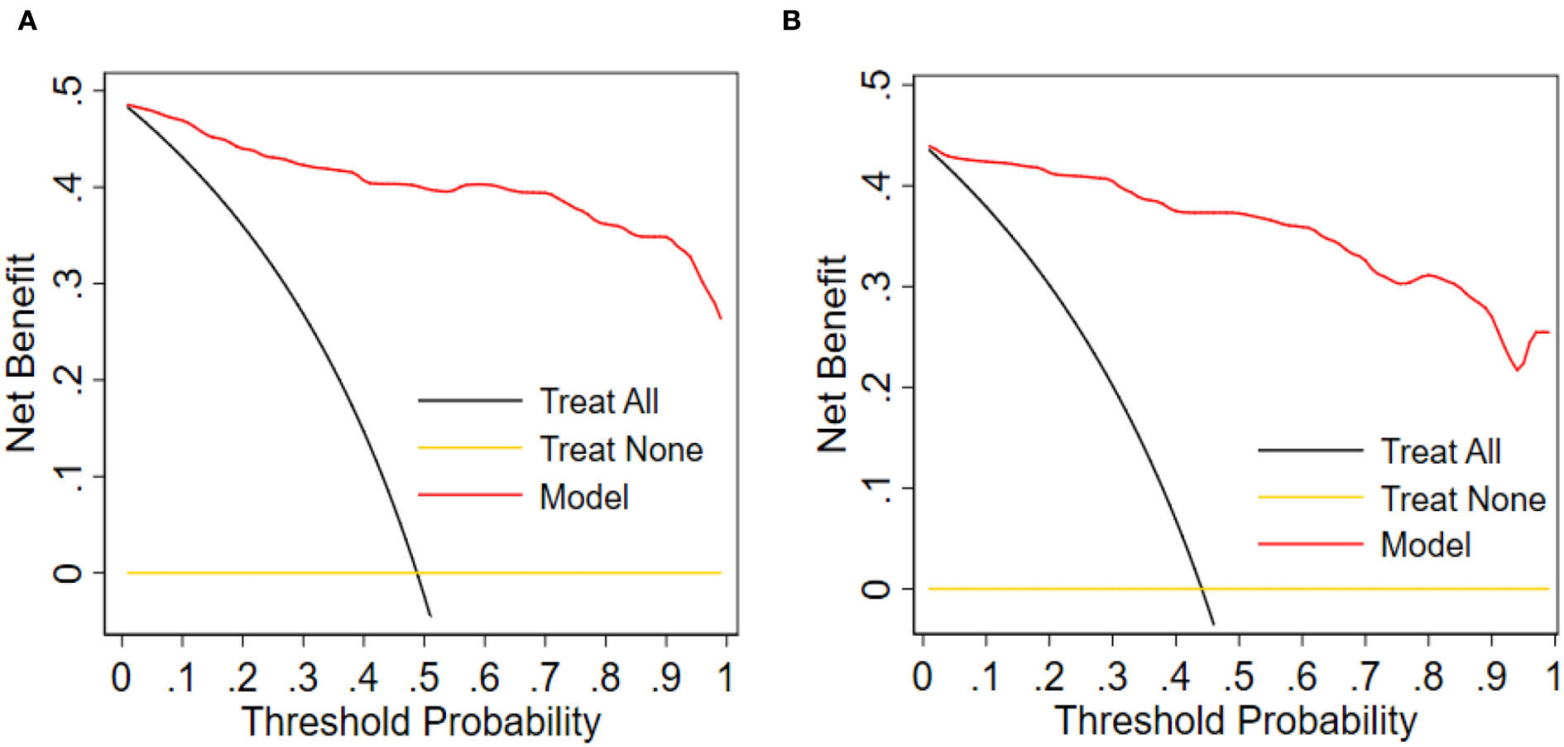
Decision curve analysis for the nomogram. **(A)** Training group. **(B)** Validation group. Obviously, the threshold probability is shown by the x-axis. The threshold probability is the value at which the expected benefit of treatment can be equivalent to the value of avoiding treatment. The y-axis measures the net benefit, computed by subtracting the rate of all patients who are false positives from the proportion of those who are truly positives and weighted by the relative risk of forgoing treatment compared with the negative consequence of the unnecessary treatment.

As demonstrated in DCA, with the threshold probabilities of 0.0 to 1.0, the application of this nomogram to predict the risk of ICM offers a higher net benefit in comparison with the “treat all” or “treat none” strategies, thus demonstrating the good clinical usefulness of the nomogram (Figures 7A,B).

### Cardiogenic death events in the follow-up period

**Figure 8:**
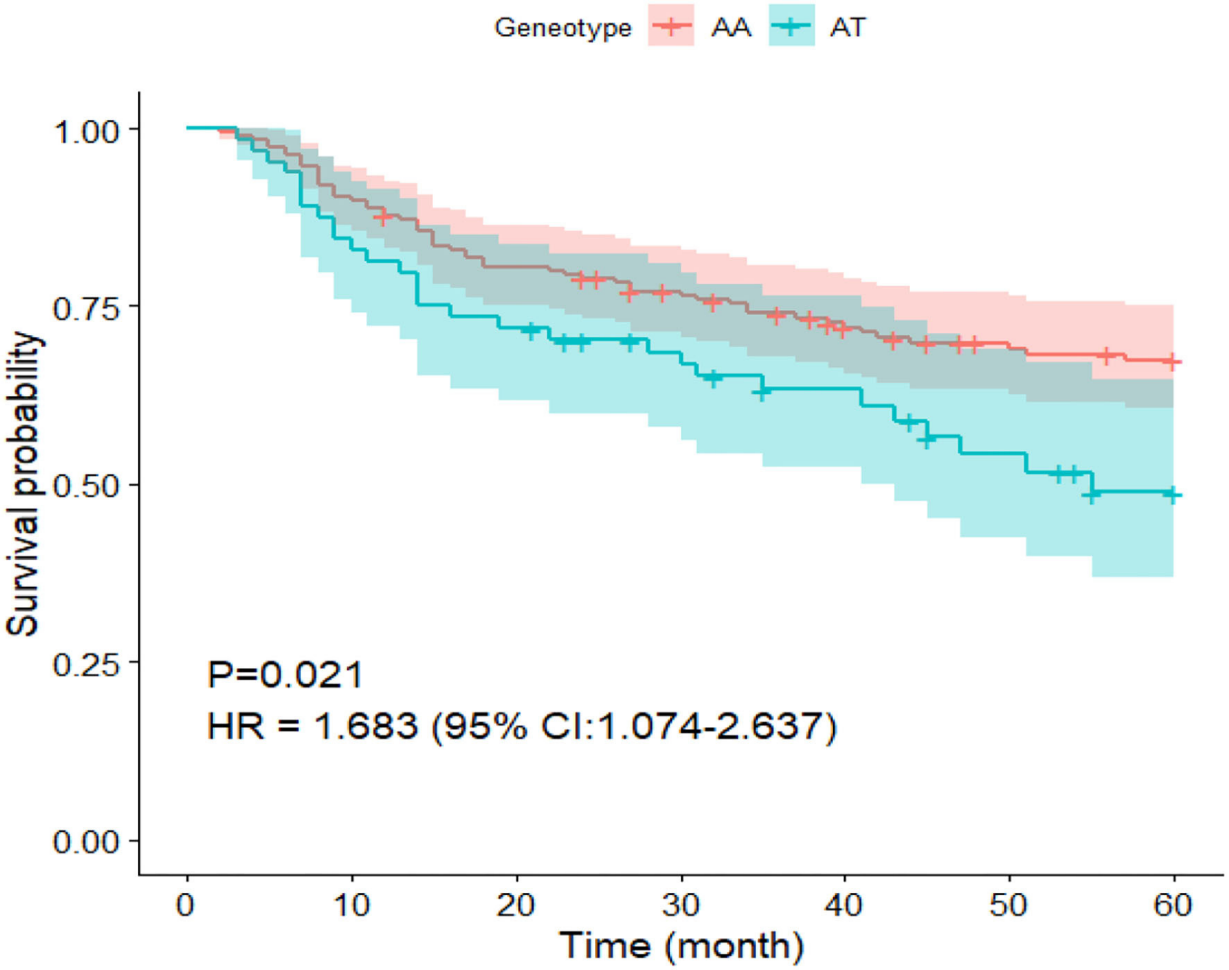
Survival curve of patients undergoing ICM carrying the AA and AT genotypes in the SNP rs5491.

During the in-hospital stay and after discharge, this study involved 252 patients diagnosed with ICM to investigate the potential influence of the *ICAM-1* gene rs5491 on the cardiogenic death rate. During the 60-month (range: 2–60 months) follow-up period, 85 patients died due to cardiogenic death (56 patients with the AA genotype and 29 with the AT genotype). According to the Kaplan–Meier curve analysis and Cox regression analysis, the risk of cardiogenic death was higher in ICM patients with the AT genotype than in those carrying the AA genotype within the follow-up phase (*P* < 0.05, HR = 1.68; 95% CI: 1.074–2.637) (Figure 8).

## Discussion

In the present single-center case-control study, the following variables were determined as risk factors for ICM: age, smoking, diabetes, LDL-C, hemoglobin, NT-proBNP, ejection fraction, and rs5491. We found a correlation between the SNP rs5491 of the *ICAM-1* gene and ICM by studying 252 patients with angiographically determined ICM and 280 control subjects. In addition, the AA genotype frequency of the rs5491 variant in the *ICAM-1* gene was lower in ICM patients than in those with the AT genotype. Individuals carrying the AT variant were shown to have a 5.816-fold higher frequency of ICM than those carrying the AA variant. In accordance with the findings of the logistic regression analysis, we proposed a predictive nomogram model (Figure 4) to identify patients with a high possibility of developing or who have already developed ICM. Moreover, during the 60-month follow-up period, ICM patients carrying the AT genotype were more likely to have cardiogenic death than those carrying the AA genotype (Figure 8).

Although a previous study (21) found a correlation between the level of soluble ICAM-1 (sICAM-1) and the severity of atherosclerosis and also described that the inhibition of ICAM-1 expression could postpone the progression of atherosclerosis in apolipoprotein E-knockout mice, no study has proved the nomogram of association between the polymorphism of rs5491 in the ICAM-1 gene and coronary artery disease or ICM. Thus, this is a novel finding and has a significant role in predicting ischemic cardiomyopathy more accurately.

**Figure 9:**
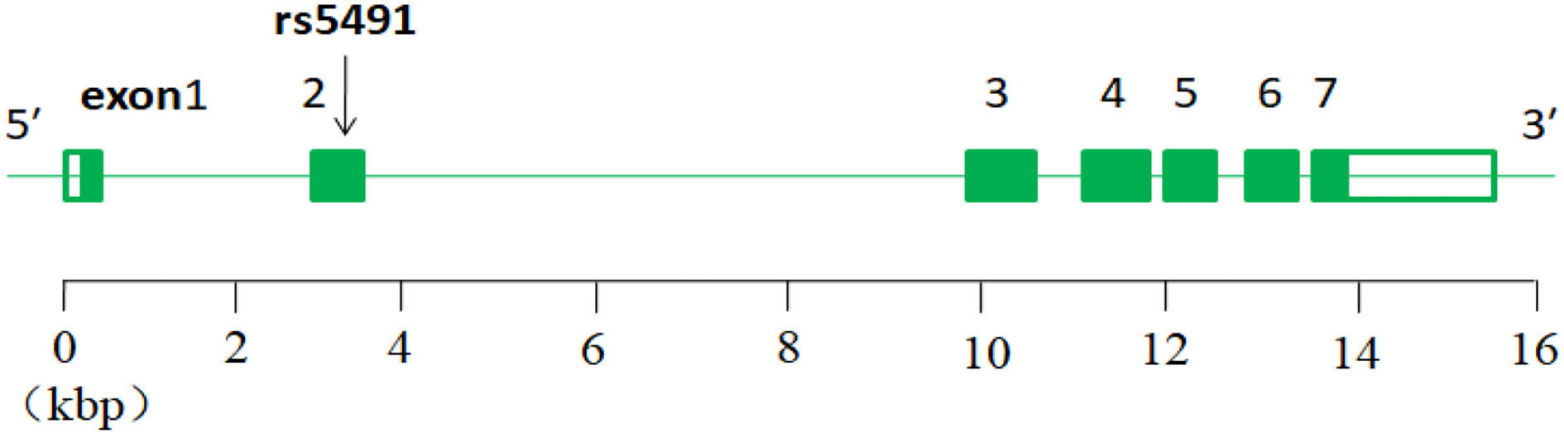
Diagram of ICAM-1 gene and location of rs5491.

The intercellular adhesion molecule-1 (ICAM-1) gene is located on chromosome 19 (Chr19:10,271,120–10,286,615;15.495 kbp) and consists of seven exons separated by six introns (22), and the rs5491 SNP is located on the ICAM-1 gene (exon2) (Figure 9).

It has an A allele gene and an AA wild-type gene. In the world population, the frequency of allele gene A is 0.985219, T is 0.014781, and in the Asian population, A is 0.9311, T is 0.0689. The wild-type gene AA can mutant to AT and TT genotypes; the SNP rs5491 (K56M in exon2) has the role of synthetic amino acid in homo sapiens. When the mutant (from AA to AT) happened on it, the role of the synthetic lysine acid changed to become a start codon (K56M) (22). It is probably the main cause of the development of the ICM.

Although we find a correlation between the polymorphism of rs5491 and ICM, the causal relationship regarding whether the rs5491 mutation results in ICM or whether the rs5491 mutation occurs after the ICM through a self-protective response of ICM patients and its pathological mechanisms remains unclear. The study participants were people living in Xinjiang, China, whose dietary eating habits included enjoyment of meat, especially fatty meat, and high-salt and high-fat foods. As recognized in earlier studies, eating habits such as these are the leading risk factor for CAD. As shown in the nomogram (Figure 4), except for the rs5491 mutation, the other factors, including age, smoking, diabetes, and LDL-C, were also risk factors for ICM, with a greater contribution to diagnosing ICM than the rs5491 mutation. Except for the rs5491 mutation, these factors have been accepted as the independent risk factors for CAD and ICM in previous studies. Thus, the current work could not determine the causal correlation between the rs5491 mutation and ICM. This implies that this correlation's causal relationship and pathological mechanisms should be clarified in further studies.

Regardless of the causal relationship, according to the results of the present study, it is clear that the rs5491 SNP correlates with the morbidity of ICM, and it has a diagnostic value for ICM patients unwilling to undergo coronary angiography to diagnose the condition according to the gold standard.

In the medical community, nomograms have gained widespread acceptance as reliable prognostic tools. User-friendly digital interfaces are essential to the success of nomograms, which improve accuracy, facilitate a better understanding of prognosis, and ultimately aid in better clinical decision-making and outcome prediction for CVDs (23, 24). This work was the first attempt to establish a nomogram in ICM.

To explore the potential predictive value of the *ICAM-1* gene variants, we attempted to establish a nomogram to compare *ICAM-1* rs5491 genotypes, age, smoking, diabetes, hemoglobin, LDL-C, left ventricular ejection fraction, and NT-proBNP to predict the risk of ICM. We validated this predictive model based on its discrimination ability, calibration ability, and DCA. The developed nomogram showed good discriminative ability according to the AUC value of ROC, as illustrated in Figures 5A,B). We then plotted the calibration curve (Figures 6A,B). The calibration curve of the nomogram model and the result of the Hosmer-Lemeshow test (*P*_training/group =_ 0.937, *P*_validation/group =_ 0.910) showed that the predicted probability was highly consistent with the actual probability. The DCA is a new test for assessing nomogram models (25). As shown in Figures 7A,B), the DCA demonstrated that if the threshold probability was within the range of 0.0 to 1.0, adopting this ICM nomogram to predict ICM risk provided more benefits than the use of the “treat all” or “treat none” strategies, thus showing the clinical usefulness of the nomogram. In this way, the net benefit was comparable with some overlaps by adopting the ICM predictive nomogram.

Regarding the 60-month follow-up data, the Kaplan–Meier analysis and Cox regression analysis showed that ICM patients who carried the AT genotype were more likely to have cardiogenic death than those carrying the AA genotype (Figure 8).

Nevertheless, this work still has certain limitations. Initially, this was a single-hospital study with a small sample size. Therefore, future research should include a larger sample size and multicenter cohorts to verify our findings. Second, because the model was validated internally, this study's generalizability (external validity) remains unclear. Third, the study participants were residents of China; therefore, there is a need to confirm the results of this study for populations living in other countries. Fourth, in the result of this study, except for rs5491, several variables recognized as the risk factors for ICM in the previous study are regarded as the predicted factors for ICM. These factors may be confounded to accurately describe the relevance between rs5491 and ICM. We found that the correlation was initially between rs5491 polymorphism and ICM. In our further study, we need to control the other variables recognized in an earlier study by matching the variables in the case group and control group and describing the relevance more accurately between rs5491 polymorphism and ICM.

In conclusion, our study found a correlation between the rs5491 gene variants in the *ICAM-1* gene and the frequency of having ICM. The AT genotype carriers had a higher frequency of ICM; individuals with a mutant rs5491 AT genotype showed a 5.816-fold higher frequency of ICM than those carrying the AA genotype. ICM patients carrying the AT genotype have worse clinical outcomes in terms of cardiogenic death than those carrying the AA genotype. We developed an early predictive model incorporating clinical variables and *ICAM-1* gene variation; this model was beneficial as a predictive model for identifying patients at risk of developing ICM, which will contribute to better management and treatment of patients with ICM.

## Data availability statement

The datasets presented in this study can be found in online repositories. The names of the repository/repositories and accession number(s) can be found in the article/supplementary material.

## Ethics statement

The studies involving human participants were reviewed and approved by Ethics Committee of the First Affiliated Hospital of Xinjiang Medical University. The patients/participants provided their written informed consent to participate in this study.

## Author contributions

All authors listed have made a substantial, direct, and intellectual contribution to the work and approved it for publication.

## Funding

This study was funded by the State Key Laboratory of Pathogenesis, Prevention and Treatment of High Incidence Disease in Central Asia Fund, Xinjiang Medical University project (No.SKL-HIDCA-2019–18).

## Conflict of interest

The authors declare that the research was conducted in the absence of any commercial or financial relationships that could be construed as a potential conflict of interest.

## Publisher's note

All claims expressed in this article are solely those of the authors and do not necessarily represent those of their affiliated organizations, or those of the publisher, the editors and the reviewers. Any product that may be evaluated in this article, or claim that may be made by its manufacturer, is not guaranteed or endorsed by the publisher.
